# Self-assessed tactical skills in tennis players: Psychometric evaluation of the Tactical Skills Questionnaire in Tennis

**DOI:** 10.3389/fspor.2022.988595

**Published:** 2022-09-27

**Authors:** Nikki S. Kolman, Barbara C. H. Huijgen, Marieke J. G. van Heuvelen, Chris Visscher, Marije T. Elferink-Gemser

**Affiliations:** ^1^Center for Human Movement Sciences, University Medical Center Groningen, University of Groningen, Groningen, Netherlands; ^2^Knowledge Center for Sport and Physical Activity, Ede, Netherlands; ^3^Department of Psychology, University of Groningen, Groningen, Netherlands

**Keywords:** racket sports, tennis, Tactical Skills Questionnaire in Tennis (TSQT), principal component analysis, talent development, performance

## Abstract

To our knowledge, no feasible, valid and reliable instrument exists to examine tactical skills over the course of multiple training and game situations in tennis yet. Therefore, the aim of this study was to develop and evaluate the psychometric properties of the Tactical Skills Questionnaire in Tennis (TSQT). The TSQT is a new instrument with closed-ended questions designed to examine tactical skills in tennis players. Participants were 233 competitive tennis players (age: 17.06 ± 4.74 years) competing on national or regional levels. With a principal component analysis (PCA) we identified four theoretically meaningful subscales for the 31-item TSQT: “Anticipation and positioning,” “Game intelligence and adaptability,” “Decision-making,” and “Recognizing game situations” and confirmed them with a confirmatory factor analysis (CFA) (χ^2^ = 527.02, df = 426, *p* < 0.001, CFI = 0.93, RMSEA = 0.045, SRMR = 0.079). Internal consistency was good, with Cronbach's alpha of 0.89 for the entire scale and McDonald's omega ranging from 0.69 to 0.78 for the separate subscales. A subsample of 57 players completed the TSQT twice to assess test-retest reliability. Absolute test-retest reliability of the subscales was good with no significant differences in mean scores between test and retest (*p* > 0.05). Relative test-retest reliability was moderate with ICC values ranging from 0.65 to 0.71. National players outperformed regional players on the subscales “Game intelligence and adaptability,” “Decision-making,” and “Recognizing game situations” (*p* < 0.05), and there was a trend toward significance for “Anticipation and positioning” (*p* = 0.07). This study supported the psychometric properties of the TSQT. Evaluating tactical skills with the TSQT provides players, coaches and other professionals with insight in players' self-assessed tactical skills over the course of multiple training and game situations. It creates the opportunity for players to reflect on their skills and detect personal development areas with their coach. We advise to use this information as input for tailor-made training programs.

## Introduction

Outstanding tactical skills are requisite for elite performance in many sports (1–4). At the highest performance level in dynamic open-ended sports like tennis, players must often make quick and accurate tactical decisions (5). In temporally constrained situations, they must detect and use contextual and kinematic information to anticipate the opponent's intentions. Specific sources of contextual information, including shot sequence and the position of the players on the court, facilitate player anticipation (6). Some have suggested that these contextual sources include the minimal required information needed for successful anticipation, and that the later emergence of kinematic information from the opponent's actions around ball-racket contact may be confirmatory (7, 8). In other words, as postural cues from the opponent become available, the number of options for responding appropriately may decrease to permit the emergence of an option with high success likelihood. Elite tennis players have been found to be better at detecting and using contextual and kinematic information than less skilled players, resulting in their superior anticipation and decision-making skills compared to players with lower performance levels (9). For example, they have a greater ability to put pressure on their opponents by choosing responses that are more likely to compromise the opponent's actions (e.g., force the opponent to move or play to their weaker side) (10). Players' positions on the court are crucial, as an optimal position enhances court coverage and enables an effective response to the opponent's most likely stroke direction. Not surprisingly, game intelligence has been considered essential for tennis performance, and it is often defined as the ability to “read the game” and act accordingly (11). As all these tactical skills (e.g., anticipation, decision-making, positioning, and game intelligence) must be well developed to meet the game's competitive demands, monitoring them is important to assist player development. This is particularly relevant for talented youth players aiming to reach the elite level. Still, no instrument is available to assess these skills over the course of multiple tennis training and game situations.

A feasible instrument to gather information on players' tactical skills is the Tactical Skills Inventory for Sports (12). This self-report questionnaire measures invasive game player's accumulated know-how on their tactical skills over a prolonged period of time, independent of their shape of the day or their opponent. It contains scales for declarative knowledge describing “knowing what to do” and procedural knowledge relating to “doing it”. Research in field hockey players revealed that elite players scored higher than amateur players on both self-assessed declarative and procedural knowledge (13). However, within a group of elite players the selection of an appropriate action within the context of the game, i.e., procedural knowledge, seems to differentiate more between performance levels than knowledge of the rules and goals of the game, i.e., declarative knowledge. This finding is confirmed in a study with soccer players, however, less is known regarding the game of tennis. For studying tactical skills, it is important to consider both the “quality” and “quantity” of players' tactical skills. Quality is inferred from the players' excellence in the demonstrated tactical skills and quantity refers to how often players display their tactical skills. Both factors may determine match outcome. For instance, the performance depends on players' ability to make the right decision about the next stroke. Thus, the *quality* of this action affects match performance. In addition, players who make the right decision about their next stroke more often will ultimately outperform players who occasionally make the right decision. This means that the outcome of a match also hinges on the *quantity* of a players' ability to make the right decision at the right time.

To our knowledge, no feasible, valid and reliable instrument exists to examine procedural knowledge (e.g., decision-making, anticipation, positioning, and game intelligence) over the course of multiple training and game situations in tennis yet. Such instrument provides players, coaches and other professionals with insight in players' self-assessed tactical skills. It creates the opportunity for players to reflect on their skills and together with the coach to detect personal development areas. As such, it can provide relevant input for the content of training programs. Considering the relevance of assessing these skills in tennis, the aim of this study is to develop and evaluate the psychometric properties of the Tactical Skills Questionnaire in Tennis (TSQT) with a sample of competitive tennis players. Specifically, the purpose is to assess its content validity, construct validity, internal consistency, test-retest reliability and discriminative validity.

## Materials and methods

We conducted this study in seven phases: (a) questionnaire design and construction, (b) exploration of the readability and comprehension of questionnaire items, (c) identification of components with principal component analysis (PCA), (d) verification of the component model with confirmatory factor analysis (CFA), (e) examination of internal consistency, (f) evaluation of test-retest reliability, and (g) assessment of discriminative validity. We used the COSMIN Study Design Checklist for Patient-Reported Outcome Measurement Instruments for reporting on these procedures (14).

### Ethical considerations

We obtained ethical approval for this research protocol (PSY-1819-S-0262) from the Psychology Department of the University of Groningen (Groningen, Netherlands, September 19th, 2019), and we obtained advanced written informed consent or assent from all players and advanced written informed consent from parents or legal guardians of all players under 16 years of age (the legal age for giving consent in the Netherlands).

### Participants

The study's inclusion criteria required participants to be healthy volunteers, between 10 and 35 years of age, who had both competitive tennis experience and sufficient proficiency in speaking, reading and writing Dutch to take this questionnaire. Based on common sample size recommendations for conducting factor analysis, our aim was to recruit at least 200 participants (15). We recruited participants from different tennis clubs in the Netherlands and the Royal Dutch Lawn Tennis Association (KNLTB). Our total participant sample included 233 competitive tennis players (160 males, 73 females; *M* age = 17.06, *SD* = 4.74 years). The average number of training hours per week (including both tennis training and strength and conditioning training) ranged between 0 and 3 h per week for 26.7% of the sample. For 18.1% of the sample, the average training per week was at least 3 h. For 19.4, 17.2, and 18.5% of the sample, the average training per week was at least 6, 9, and 12 h, respectively.

### Development of the Tactical Skills Questionnaire in Tennis

The aim of the first two phases of this research was to ensure acceptable content validity of the TSQT. In other words, we first sought to confirm that the questionnaire adequately covered all relevant tactical skills to be measured (16). Following advice from Artino et al. (17), we began with a literature review that helped to operationalize the construct of tactical skills and determined whether other similar measures of tactical skills in tennis already existed. Finding no evidence of any similar instrument we designed the TSQT by using the TACSIS as an example model, reformulating various TACSIS items to be tennis-specific (12). For example, we changed an item of the TACSIS “*My positioning during a match is generally*” into “*My position on the court is*”. We adapted another item from the TACSIS “*My anticipation (thinking about proceeding actions) is*” to “*In looking ahead (thinking about my next stroke), I am*”. Next, we relied on a group of four scientists with extensive experience in research on tactical skills in sports (experience on this topic ranging from 7 to 20 years) to formulate new items for the domains of anticipation, positioning, decision-making and game intelligence. A distinction was made between the quality and quantity of these skills. The quality of tactical skills was inferred from the players' self-assessed excellence in the demonstrated tactical skills and the quantity of tactical skills was inferred from the players' self-assessed frequency of displaying the tactical skills.

In the next step we discussed the new items with an expert panel consisting of an embedded scientist, a performance manager and two highly experienced international tennis coaches of the Royal Dutch Lawn Tennis Association (KNLTB). The expert panel offered suggestions for improving the questionnaire, including adding items to assess the ability to read game situations before acting and performing. We then formulated or reformulated items to meet this need (e.g., “*I quickly see when my opponent changes the direction of the ball*”). The expert panel also indicated a need to distinguish between tactical skills when a player has a lot of response time (offensive situation), enough time (neutral situation) or not enough time (defensive situation). Again, we formulated and reformulated items to address this domain in different situations (e.g., “*In making the right decisions when my opponent is under pressure, I am:*” was developed for a situation in which players have a lot of time, the item “*In a cross rally I choose the right moment to open down the line*” was developed for a neutral situation in which players have enough time, and “*My position when I am under pressure from my opponent is:*” was developed for a defensive situation in which players do not have enough time).

In the second phase we examined the readability and comprehension of each item. We piloted a preliminary version of the questionnaire for 13 youth tennis players aged 12–14 years to check the understanding of items within the youngest age groups who would be completing the questionnaire. Players completed the questionnaire individually during tennis practice and were allowed to give comments and suggestions. We confirmed that these young participants understood all items, except two, and we reformulated these two items. Thus, the first and second phase of test development resulted in an initial 38-item TSQT, with content validity supported by the results of the literature review, expert item evaluation and pilot testing. We developed the questionnaire in Dutch and then translated it into English according to the back-translation procedure whereby one researcher with a proficiency in both languages translated the items from Dutch to English and these English items were translated back to Dutch by another bilingual translator. We compared the new translations with the original items and made several minor linguistic modifications to maintain the intended item meanings. The final questionnaire can be found in Supplementary material 1.

#### TSQT structure

The TSQT consisted of 38 items on a 5-point Likert scale. We chose an uneven-point scale with a neutral middle option to avoid forcing respondents to answer positively or negatively. To minimize response bias, we placed negative options on the left side of the scale and positive options on the right side of the scale (18). As such, the questionnaire provided two semi-negative choices: “almost never” and “sometimes” relating to questions about the quantity of skills and “very mediocre” and “mediocre” relating to questions about the quality of skills. There was one neutral option (“regularly” or “reasonable”) and two semi-positive choices (“often” and “almost always” or “good” and “very good”). To improve the reliability, we labeled all options (18). The questionnaire ended with some demographic questions about the respondent's age, gender, tennis level and training hours.

### Procedures

We administered the questionnaire to our 233 participants at different tennis clubs and the Royal Dutch Lawn Tennis Association (KNLTB) in the Netherlands. Participants completed the questionnaire individually with a researcher present. A subsample (*n* = 57) completed the questionnaire twice within 2–4 weeks. The time interval between test and retest was considered long enough to reduce the chance of participants recalling their first answers, and short enough to reduce the chance for a true change of the construct to occur (19).

### Statistical analysis

For most statistical analyses, we used the Statistical Package for the Social Sciences for Windows (SPSS, version 26; IBM Corp., Armonk, N.Y., USA). For the confirmatory factor analysis (CFA), we used LISREL for Windows, version 8.80 (20). For all significance tests, we used an α-level of 0.05. We checked normality of the data distribution for items by exploring normality plots and z-scores for skewness and kurtosis. The percentage of missing values across the 38 items varied between 0 and 2.6%. We imputed missing values with regression estimates obtained by predicting missing values with a regression of observed scores on other items. After stratification on age, gender and tennis level, we randomly allocated subjects to the group for PCA (*n* = 117) and CFA (*n* = 116).

#### Principal component analysis

In the third phase, we assessed the adequacy of sampling by Kaiser Meyer Olkin (KMO). We interpreted the KMO using the guidelines of Hutcheson and Sofroniou (21) (0.40 = minimum; 0.50–0.70 = mediocre; 0.70–0.80 = good; 0.80–0.90 = great; >0.90 superb). To determine if correlations between items were sufficiently large to perform a PCA, we used Bartlett's Test of Sphericity. We performed a PCA to examine the component structure of the 38-item questionnaire (i.e., the construct validity). Construct validity refers to whether the items of a questionnaire represent the underlying conceptual structure (22). Due to conceptual considerations, we extracted four components in the analysis. We used an oblique rotation, because all items were intended to measure the same concept and components were assumed to correlate. We deleted items with low communalities (<0.30) and/or items with low component loadings on each component (<0.30). A low communality suggests that the item has little in common with the other items and a low component loading means that the component has a weak association with the principal component score.

#### Confirmatory factor analysis

In the fourth phase, we used a CFA to verify the four-component model identified by the PCA. We estimated the relationships between the four components and between each item and the corresponding component. We also estimated the explained variance and error variance for each item. We judged the adequacy of model fit by the following fit statistics: comparative fit index (CFI), root mean square error of approximation (RMSEA), and standardized root-mean square residual (SRMR). For the CFI, we considered values of ≥0.90 as acceptable and values of ≥0.95 as good (23, 24). For the RMSEA, we interpreted values of ≤0.06 as good (24). For the SRMR, we considered values of ≤0.08 as acceptable and values of ≤0.06 as good (24–26). We also examined the chi-square value; however, the statistic is highly sensitive to sample size (27). We used modification indices and theoretical arguments to improve the model fit.

#### Internal consistency

In the fifth phase, we calculated mean item scores for each subscale of the TSQT. To assess the internal consistency of the TSQT, we determined the average inter-item correlation and McDonald's omega for each subscale and Cronbach's alpha for the total scale. In contrast to the commonly reported Cronbach's alpha, McDonald's omega makes fewer and more realistic assumptions and problems associated with inflation and attenuation of internal consistency estimations are far less likely (28). We considered an average inter-item correlation between 0.15 and 0.50 as good (29). In agreement with the guidelines of Nunnally (30) for Cronbach's alpha, we considered McDonald's omega of ≥ 0.7 as acceptable. To determine the relationships between subscales, we calculated Pearson's correlation coefficients based on mean item scores. We interpreted the strength of the relationship as weak (0.10–0.30), moderate (0.30–0.50), or strong (>0.50) (31).

#### Test-retest reliability

In the sixth phase, we assessed test-retest reliability with a subsample of 57 tennis players (34 males, 23 females; age: 18.78 ± 4.60 years). The size of the subsample corresponds with the recommended sample size of at least 50 participants for test-retest reliability (32, 33). We determined the absolute and relative reliability of the TSQT. Absolute reliability refers to the degree to which repeated measurements vary for individuals. Relative reliability refers to the ability of individuals to maintain their rank in a sample with repeated measurements (34). To estimate the absolute test-retest reliability of the TSQT, we calculated mean differences between test and retest, with 95% confidence intervals. We assessed the relative test-retest reliability by intraclass correlation coefficients (ICC) with 95% confidence intervals based on single ratings, consistency and two-way mixed-effects model. We interpreted the ICC values using the guidelines of Koo and Li (35) (<0.5 = poor; 0.5–0.75 = moderate; 0.75–0.90 = good; >0.90 = excellent).

#### Discriminative validity

In the last phase, we evaluated discriminative validity within a sample of 218 players since the competitive level of 15 players was unknown. Players were classified as national or regional according to their competitive level of performance. National players competed nationally (usually throughout the Netherlands) or internationally (usually in other countries), while regional players usually competed in their own region in the Netherlands. The sample consisted of 88 national players (54 males, 34 females; age: 15.61 ± 4.35 years) and 130 regional players (97 males, 33 females; age: 18.07 ± 4.70 years). We assessed the discriminative validity by a one-way multivariate analysis of covariance (MANCOVA) with performance level as between-subjects factor (national vs. regional) and four subscales as dependent variables, whilst controlling for age and sex as covariates. We hypothesized that national players would outperform regional tennis players on the different subscales of the TSQT.

## Results

### Principal component analysis

The KMO measure of sampling adequacy was 0.82, which was considered great, and Bartlett's test of sphericity was significant [$\chi_{\left(703\right)}^{2}$ = 1,790.28, *p* < 0.001] indicating that there was a certain redundancy between the items that could be summarized with a few components. The initial PCA yielded a four-component model that explained 42.1% of the variance. Six items with communalities of <0.30 and one item with a pattern coefficient of <0.30 were removed from the questionnaire for subsequent analysis. A second PCA was performed on the retained 31 items. Items and pattern loadings are presented in Table 1. In total, the four components accounted for 47.0% of the variance (27.6, 7.4, 6.5, and 5.5%, respectively, before rotation). The components were labeled “Anticipation and positioning” (Component 1, 10 items), “Game intelligence and adaptability” (Component 2, 6 items), “Decision-making” (Component 3, 8 items), and “Recognizing game situations” (Component 4, 7 items).

**Table 1:** Items and pattern loadings of the TSQT.

	**1**	**2**	**3**	**4**
**Quantity of tactical skills (1** **=** **almost never and 5** **=** **almost always)**				
1. I use the weak spot of my opponent		**0.542**		0.325
2. I quickly see where my opponent is serving to	**0.692**			
3. When I am under pressure from my opponent, I make the right decisions		0.359	**0.614**	
4. In a cross rally I choose the right moment to open down the line			**0.613**	
5. Before my opponent hits the ball, I move toward the right spot	**0.622**			
6. I choose the right moment to change the direction of the ball			**0.309**	0.405
7. When my opponent serves, I quickly move to the right spot	**0.449**			0.306
8. When I want to disrupt my opponent, I change the (top) spin of my balls		**0.507**	0.421	
9. I quickly see where my opponent is standing with my service				**0.755**
10. I incorporate the experiences of earlier points in my decisions		**0.400**		0.468
11. When I want to disrupt my opponent, I change the height of my balls		**0.744**		
12. Before my opponent hits a drop shot, I move forward	**0.656**			
13. When I notice that my tactical plan is not working, I quickly adjust my game		**0.316**	0.344	
14. I quickly see when my opponent changes the direction of the ball	0.420			**0.423**
15. When I am in an attacking position, I see where the open space is				**0.738**
16. When I'm at the net, I quickly see where my opponent is hitting the ball				**0.398**
**Quality of tactical skills (1** **=** **very mediocre and 5** **=** **very good)**				
17. The decisions I make about my next stroke are generally:			**0.652**	
18. In moving to the spot where my opponent serves, I am:	**0.350**			
19. In making the right decisions at the right time, I am:			**0.680**	
20. My choice from various options to score a point is generally:			**0.568**	
21. In varying my strokes at the right time, I am:			**0.654**	
22. In being at the right spot at the right time, I am:	**0.720**			
23. My game intelligence is:	**0.421**		0.327	
24. In making the right decisions when my opponent is under pressure, I am:				**0.407**
25. My position on the court is:	**0.516**			
26. In determining the depth of an incoming ball, I am:	**0.597**			
27. My position when I am under pressure from my opponent is:	**0.499**			
28. In recognizing game situations, I am:	0.382	**0.407**		
29.In quickly recognizing my opponent's weak spot, I am:		0.467		**0.547**
30. My position when I put pressure on my opponent is:				**0.613**
31. In responding to a defensive ball of my opponent, I am:			**0.592**	

Extraction method: Principal component analysis; Rotation Method: Oblimin with Kaiser Normalization.

Pattern loadings <0.30 are not displayed, pattern loadings on the allocated component for the CFA are presented in bold.

Component 1 (Anticipation and positioning) = items 2, 5, 7, 12, 18, 22, 23, 25, 26, 27.

Component 2 (Game intelligence and adaptability) = items 1, 8, 10, 11, 13, 28.

Component 3 (Decision-making) = items 3, 4, 6, 17, 19, 20, 21, 31.

Component 4 (Recognizing game situations) = items 9, 14, 15, 16, 24, 29, 30.

### Confirmatory factor analysis

The initial CFA indicated acceptable model fit for the 31-item, four-component model identified by EFA (χ^2^ = 569.94, df = 428, *p* < 0.001, CFI = 0.91, RMSEA = 0.054, SRMR = 0.083). Modification indices suggested to add a path from the item “My game intelligence is” to “Game intelligence and adaptability” to improve model fit. The item corresponds with the content of the component; therefore, this path was added. After that, the non-significant loading of the item to “Anticipation and positioning” was deleted. Furthermore, modification indices suggested to add covariances between error terms. Therefore, the covariance between three pairs of error terms was added. The final CFA resulted in an acceptable to good model fit (χ^2^ = 527.02, df = 426, *p* < 0.001, CFI = 0.93, RMSEA = 0.045, SRMR = 0.079). The TSQT includes four subscales “Anticipation and positioning” (9 items), “Game intelligence and adaptability”, (7 items), “Decision-making” (8 items), and “Recognizing game situations” (7 items).

### Internal consistency

Descriptive statistics and internal consistency of the four subscales are displayed in Table 2. Overall, the TSQT was found to be highly reliable (α = 0.89).

**Table 2:** Descriptive statistics, McDonald's omega, and average inter-item correlation coefficients of subscales of the TSQT (*n* = 233).

	***M***	***SD***	**McDonald's** **omega**	**Inter-item** **correlation**
Anticipation and positioning (9 items)	3.47	0.54	0.78	0.29
Game intelligence and adaptability (7 items)	3.55	0.57	0.69	0.25
Decision-making (8 items)	3.46	0.51	0.77	0.30
Recognizing game situations (7 items)	3.68	0.55	0.73	0.29

The relationship between subscales is shown in Table 3. The largest positive correlation was found between the subscales “Decision-making” and “Recognizing game situations” (*r* = 0.62).

**Table 3:** Pearson correlations between subscales of the TSQT.

	**Anticipation and** **positioning**	**Game intelligence** **and adaptability**	**Decision-** **making**	**Recognizing game** **situations**
Anticipation and positioning	1			
Game intelligence and adaptability	0.51^*^	1		
Decision-making	0.48^*^	0.51^*^	1	
Recognizing game situations	0.55^*^	0.50^*^	0.62^*^	1

* p < 0.01.

### Test-retest reliability

Descriptive statistics of the absolute and relative reliability of the TSQT are shown in Table 4. A value of 0 was within the 95% confidence interval of the mean differences between test (T1) and retest (T2), supporting the absolute reliability of the TSQT. Moderate relative reliability was observed for the subscales “Anticipation and positioning” (ICC = 0.66), “Game intelligence and adaptability” (ICC = 0.65), “Decision-making” (ICC = 0.71), and “Recognizing game situations” (ICC = 0.69).

**Table 4:** Test-retest reliability for each subscale of the TSQT (*n* = 57).

	***M*** **±** ***SD***	***M*** **±** ***SD***	***M*** **±** ***SD***	**SE**	**95% CI**	**ICC**	**95% CI**
	**T1**	**T2**	**T1–T2**	**T1–T2**	**T1–T2**		**ICC**
Anticipation and positioning	3.42 ± 0.52	3.49 ± 0.51	−0.06 ± 0.43	0.06	−0.180–0.050	0.658	0.483–0.783
Game intelligence and adaptability	3.52 ± 0.54	3.51 ± 0.58	0.02 ± 0.48	0.06	−0.112–0.143	0.652	0.473–0.780
Decision-making	3.33 ± 0.48	3.38 ± 0.49	−0.09 ± 0.37	0.05	−0.185–0.012	0.703	0.544–0.814
Recognizing game situations	3.60 ± 0.60	3.51 ± 0.60	0.11 ± 0.47	0.06	−0.014–0.238	0.685	0.519–0.802

M ± SD of T1–T2 = mean difference between the score for the first and second measurement; SE of T1–T2 = standard error of the mean difference; 95% CI T1–T2 = 95% confidence interval for the mean difference; ICC, intraclass correlation coefficient; 95% CI for ICC = 95% confidence interval for intraclass correlation coefficient.

### Discriminative validity

Table 5 shows descriptive statistics of national and regional players for each subscale. One-way MANCOVA showed a difference between national and regional players on the combined dependent variables after controlling for age and sex, *F*_(4,211)_ = 5.245, *p* < 0.001; Wilk's Λ = 0.910, partial η^2^ = 0.090. Follow-up analyses showed that national players scored higher than regional players on the subscales “Game intelligence and adaptability” (*p* < 0.001), “Decision-making” (*p* < 0.001) and “Recognizing game situations” (*p* < 0.01), and there was a trend toward significance for “Anticipation and positioning” (*p* = 0.07).

**Table 5:** Descriptive statistics of national and regional players for each subscale of the TSQT (*n* = 218).

	**National (*****n*** = **88)**		**Regional (*****n*** = **130)**					
	***M***	***SD***	***M***	***SD***	***F***	***df***	***p***	**ηp2**
Anticipation and positioning	3.53	0.57	3.42	0.53	3.309	1, 214	0.070	0.015
Game intelligence and adaptability	3.69	0.56	3.42	0.57	13.155	1, 214	<0.001	0.058
Decision-making	3.64	0.51	3.32	0.48	16.139	1, 214	<0.001	0.070
Recognizing game situations	3.83	0.53	3.56	0.54	9.975	1, 214	0.002	0.045

## Discussion

Our aim in the present study was to develop and evaluate the psychometric properties of the TSQT with a sample of competitive tennis players. Findings of this study supported its content validity, construct validity, internal consistency, test-retest reliability and discriminative validity.

We affirmed content validity by the results of the literature review and item evaluation by the expert panel. Previous studies have shown the relevance of tactical skills for elite tennis players (6, 9, 36). In the common categorization of tactical skills based on declarative or procedural knowledge, it appeared that procedural knowledge discriminated best between the more and the less successful field hockey and soccer players (13, 37). To avoid a ceiling effect in the answers for tennis players at the highest level, we specifically developed items about procedural knowledge, i.e., “doing it” in tennis. We adapted numerous items for procedural knowledge from the TACSIS and applied them to tennis. We formulated novel items around the construct of tactical skills. All items were checked by the expert panel and four authors of this study who confirmed that they represent tactical skills in tennis.

With the PCA and CFA, we omitted seven items from the original 38-item questionnaire because they made insufficient contribution to the component (i.e., the pattern loading was too low) or they loaded on the non-hypothesized component. For example, we deleted the item “*My choice to lob or pass when my opponent is at the net is:*” due to a low pattern loading. The item touches on more than one issue (i.e., choice to lob and choice to pass), but leaves room for only one response. Respondents might have understood this double-barreled item differently, resulting in a weak influence on the component. Final analyses resulted in a 31-item TSQT, composed of four subscales: “Anticipation and positioning” (9 items), “Game intelligence and adaptability” (7 items), “Decision-making” (8 items), and “Recognizing game situations” (7 items). The four subscales of the TSQT are considered to represent important domains of tactical skills in tennis, supporting the construct validity of the TSQT. Nevertheless, the four-component model structure explained merely 47% of variance in the instrument, suggesting that tactical skills may be affected by a broader range of factors than are assessed within this scale.

We confirmed the internal consistency of the TSQT by average inter-items correlations from 0.25 to 0.30, Cronbach's α of 0.89 and McDonald's ω ranging between 0.69 and 0.78 for the separate subscales. These coefficients were similar to those reported for the TACSIS (12). The high internal consistency found in the present study clearly demonstrates that the items of the TSQT measure the same concept. This is supported by the positive correlations between the subscales. Moreover, the subscales of the TSQT were absolutely and relatively stable over time, indicating that the TSQT is a reliable questionnaire for examining these skills in competitive tennis players. The time interval of 2–4 weeks between test and retest was considered long enough to make the players forget their answers from the first test, and short enough for players to improve their tactical skills. The ICC values for the subscales were between 0.65 and 0.71, indicating that the TSQT meets moderate to acceptable levels of reliability for application in a group of competitive tennis players (35). The ICC values were similar to those reported in youth hockey players for the subscales of the TACSIS (ICC 0.60–0.88) (12).

We largely confirmed the discriminative validity of the TSQT by differences between national and regional players on the TSQT and the subscales “Game intelligence and adaptability,” “Decision-making,” and “Recognizing game situations”. These results are in line with the results of a systematic review showing that players with higher performance levels display superior tactical skills than players whose performance levels are lower (9). However, national players did not outperform regional players on the subscale “Anticipation and positioning”, although this finding almost reached statistical significance (*p* = 0.07), but the effect size was small at 0.015, measured by partial-eta squared. One possible explanation for the non-significant finding could be that the sample size was too small, and the study might have been underpowered to detect differences between performance levels for each subscale of the TSQT. It could also be possible that differences in performance level between the national and regional youth players might have been too small to discriminate performance levels for all subscales. An alternative explanation might be that the items underlying the subscale “Anticipation and positioning” were not precise enough to detect differences at the group level.

There are several practical applications of the TSQT. Evaluating self-assessed tactical skills in tennis players provides players, coaches and other professionals with insight in players' tactical skills. They can use the TSQT to reflect on player's self-assessed strengths and weaknesses. This can open the discussion about the content of the training programs and designing tailor-made exercises to optimize performance development. With the TSQT, one can specifically target areas for development such as working on, for example, “choosing the right moment to open down the line in a cross rally”. If it becomes clear that a player assesses him- or herself low on this item from the subscale “Decision-making”, the coach can create a training environment in which the player is challenged in this situation specifically. Focusing on a player's strengths is crucial as well, so that players can learn to use their strengths in order to win matches. The TSQT can also assist in making players aware of their development areas and stimulate their self-regulated learning. By having them self-assess their tactical skills, they are stimulated to share and take responsibility for their own developmental process. Various studies among talented athletes have shown the value of well-developed self-regulatory skills, such as reflection, for performance and performance development (38–40). It is essential to realize that due to the characteristics of the TSQT, being a self-report measure, it is not suitable for selection purposes. Players may give socially desirable answers if they feel that reporting less-developed domains of tactical skills may have adverse effects for them, such as decreasing their chances for selection. This makes the comparison between individual players based on their responses on the TSQT questionable.

The advantage of a self-report measure such as the TSQT lies not only in its value for creating moments of reflection of players. The questionnaire also opens up the opportunity to assess large groups in a relatively easy way and derive rich contextual information. In addition, since it taps into the accumulated know-how of players and covers multiple training and game situations, it is less influenced by a player's shape of the day or opponent compared to so called “objective” measures of tactical skills. Objective methods of assessing tactical skills include measures that directly assess observed performance in one or more tactical domains. These methods may use a variety of metrics, such as number of eye fixations and correct responses for anticipating groundstroke type and direction [see for a review (9)]. Although no gold standard for objective tactical skills assessment has emerged, popular measures include temporal occlusion paradigms, stick-figure stimulations and observational instruments (5, 41, 42). Despite it can be argued that objective measures have the advantage for obtaining unbiased, reliable data, these measures merely focus on one or a limited number of aspects of tactical performance which are observed during a limited number of training sessions or games. This may be one of the reasons why in a study on soccer players no statistically significant relationship between self-assessed tactical skills as measured by the TACSIS and objective tactical performance during small sided games has been found (43). In addition, not seldom, objective measures of tactical skills are expensive and time-consuming. This makes them less suitable if one aims to assess tactical skills over the course of multiple training and game situations in large groups.

Several strengths and weaknesses of this study are acknowledged. One strength of the current study was that it focused on both the quantity and quality of tactical skills. By gaining insight in both factors, an appropriate picture can be obtained from player's tactical skills. The importance of both factors seems to be confirmed by the fact that the ratio of the remaining items in terms of quantity and quality is similar after the PCA and CFA as in the initial 38-items questionnaire. A weakness of the study was related to the relatively small sample size for PCA and CFA. The literature about factor analysis provides a wide range of rough guidelines regarding an adequate sample size. Most of these guidelines consistently advocate for an absolute minimum sample size to obtain decent factor solutions, ranging from an ideal sample size of at least 50–1,000 participants (15, 44, 45). Other studies recommend a minimum sample size from 3 to 20 times the number of items (15). The sample size of this study is within these recommended ranges, but near the required minimum (15, 44, 45). However, for most of these recommendations there is little empirical evidence. In addition, the adequacy of sampling was supported with the KMO above 0.8. Moreover, Barlett's test of sphericity was significant (*p* < 0.001), indicating that it was reasonable to proceed with PCA even considering the small sample size. The application of the TSQT in other countries, cultures, performance groups, age categories and racquet sports require the verification of the conclusions in the current sample, consisting of competitive tennis players from the Netherlands. To improve feasibility, it should be examined if the psychometric properties of the TSQT are maintained if the scale consists of fewer items measuring the same construct. Future research should focus on assessing tactical skills with the TSQT longitudinally to detect any improvements in tactical skills over time due to a training program. Moreover, it would be interesting for further studies to assess tactical skills in large groups to define benchmarks per age category and males and females separately.

In conclusion, findings from this study provide coaches and other professionals with a valid and reliable questionnaire for assessing tactical skills in competitive tennis players. Evaluating tactical skills with the TSQT provides players, coaches and other professionals with insight in players' self-assessed tactical skills over the course of multiple training and game situations. It creates the opportunity for players to reflect on their tactical skills and detect personal development areas with their coach. It is advised to use this information as input for tailor-made training programs.

## Data availability statement

The original contributions presented in the study are included in the article/Supplementary materials, further inquiries can be directed to the corresponding author.

## Ethics statement

The studies involving human participants were reviewed and approved by Psychology Department of the University of Groningen. Written informed consent to participate in this study was provided by the participants' legal guardian/next of kin.

## Author contributions

NK, ME-G, BH, and CV contributed to the study conception and design. NK collected the data and wrote the first draft of the manuscript. NK and MH analyzed the data. NK, MH, ME-G, BH, and CV reviewed and edited previous versions of the manuscript, read, and approved the final version of the manuscript. All authors contributed to the article and approved the submitted version.

## Conflict of interest

The authors declare that the research was conducted in the absence of any commercial or financial relationships that could be construed as a potential conflict of interest.

## Publisher's note

All claims expressed in this article are solely those of the authors and do not necessarily represent those of their affiliated organizations, or those of the publisher, the editors and the reviewers. Any product that may be evaluated in this article, or claim that may be made by its manufacturer, is not guaranteed or endorsed by the publisher.
