# Supplementary material for: Self-assessed tactical skills in tennis players: Psychometric evaluation of the Tactical Skills Questionnaire in Tennis
Source: Front Sports Act Living. 2022 Sep 27;4:988595. doi: 10.3389/fspor.2022.988595 (PMC9552173; doi:10.3389/fspor.2022.988595)
Supplement: Supplementary file 1 [file Data_Sheet_1.docx]

**Tactical Skills Questionnaire in Tennis**

This questionnaire is about your tactical skills. The first 16 questions are about how **often** you make certain decisions, how **often** you recognize situations or how **often** you are in the right place. You can choose from 5 answers options. Choose the answer that best **fits** each description provided. There are **no** right or wrong answers.

You can choose from the following answers:

- **Almost never** = if you **almost never** do this, or if this statement does **not apply** to you
- **Sometimes** = if you **sometimes** do this, or if this statement does **a little** **apply** to you
- **Regularly** = if you **regularly** do this, or if this statement does **regularly apply** to you
- **Often** = if you **often** do this, or if this statement does **often apply** to you
- **Almost always** = if you **almost always** do this, or if this statement does **almost always** **apply** to you.

Example:

|  | **Answer options** | | | | |
| --- | --- | --- | --- | --- | --- |
|  | Almost never | Sometimes | Regularly | **Often** | **Almost always** |
| I quickly see where my opponent is serving to | О |  | О | О | О |

|  | **Answer options** | | | | |
| --- | --- | --- | --- | --- | --- |
|  | **Almost never** | **Soms** | **Regularly** | **Often** | **Almost always** |
| 1. I use the weak spot of my opponent | О | О | О | О | О |
| 1. I quickly see where my opponent is serving to | О | О | О | О | О |
| 1. When I am under pressure from my opponent, I make the right decisions | О | О | О | О | О |
| 1. In a cross rally I choose the right moment to open down the line | О | О | О | О | О |
| 1. Before my opponent hits the ball, I move towards the right spot | О | О | О | О | О |
| 1. I choose the right moment to change the direction of the ball | О | О | О | О | О |
| 1. When my opponent serves, I quickly move to the right spot | О | О | О | О | О |
| 1. When I want to disrupt my opponent, I change the (top) spin of my balls | О | О | О | О | О |
| 1. I quickly see where my opponent is standing with my service | О | О | О | О | О |
| 1. I incorporate the experiences of earlier points in my decisions | О | О | О | О | О |
| 1. When I want to disrupt my opponent, I change the height of my balls | О | О | О | О | О |
| 1. Before my opponent hits a drop shot, I move forward | О | О | О | О | О |
| 1. When I notice that my tactical plan is not working, I quickly adjust my game | О | О | О | О | О |
| 1. I quickly see when my opponent changes the direction of the ball | О | О | О | О | О |
| 1. When I am in an attacking position, I see where the open space is | О | О | О | О | О |
| 1. When I'm at the net, I quickly see where my opponent is hitting the ball | О | О | О | О | О |

The next 15 questions are about how **good** you make certain decisions, how **good** you recognize situations or how **good** your position is. You can choose from 5 answers options. Choose the answer that best **fits** each description provided. There are **no** right or wrong answers.

You can choose from the following answers:

- **Very mediocre** = if you do this **very mediocrely**, or if this statement does **not apply** to you
- **Mediocre** = if you do this **mediocrely**, or if this statement does **a little apply** to you
- **Reasonable** = if you do this **reasonably**, or if this statement does **apply reasonably** to you
- **Good** = if you do this **well**, or if this statement does **apply well** to you
- **Very good** = if you do this **well**, or if this statement does **apply completely** to you

Example:

|  | **Answer options** | | | | |
| --- | --- | --- | --- | --- | --- |
|  | Very mediocre | Mediocre | Reasonable | Good | Very good |
| In determining the depth of an incoming ball, I am: | О |  | О | О | О |

|  | **Answer options** | | | | |
| --- | --- | --- | --- | --- | --- |
|  | **Very mediocre** | **Mediocre** | **Reasonable** | Good | Very good |
| 1. The decisions I make about my next stroke are generally: | О | О | О | О | О |
| 1. In moving to the spot where my opponent serves, I am: | О | О | О | О | О |
| 1. In making the right decisions at the right time, I am: | О | О | О | О | О |
| 1. My choice from various options to score a point is generally: | О | О | О | О | О |
| 1. In varying my strokes at the right time, I am: | О | О | О | О | О |
| 1. In being at the right spot at the right time, I am: | О | О | О | О | О |
| 1. My game intelligence is: | О | О | О | О | О |
| 1. In making the right decisions when my opponent is under pressure, I am: | О | О | О | О | О |
| 1. My position on the court is: | О | О | О | О | О |
| 1. In determining the depth of an incoming ball, I am: | О | О | О | О | О |
| 1. My position when I am under pressure from my opponent is: | О | О | О | О | О |
| 1. In recognizing game situations, I am: | О | О | О | О | О |
| 1. In quickly recognizing my opponent's weak spot, I am: | О | О | О | О | О |
| 1. My position when I put pressure on my opponent is: | О | О | О | О | О |
| 1. In responding to a defensive ball of my opponent, I am: | О | О | О | О | О |
